# A chromosomal reference genome sequence for the northern house mosquito, *Culex pipiens* form *pipiens*, Linnaeus, 1758

**DOI:** 10.12688/wellcomeopenres.23767.1

**Published:** 2025-02-26

**Authors:** Jenny C Hesson, Yuki Haba, Carolyn S McBride, Edel Sheerin, Thomas C Mathers, Michael Paulini, Damon-Lee B Pointon, James W Torrance, Cibin Sadasivan Baby, Jonathan M.D. Wood, Wellcome Sanger Institute Scientific Operations: Sequencing Operations, Wellcome Sanger Institute Tree of Life Core Informatics team, Shane A McCarthy, Mara K N Lawniczak, Alex Makunin

**Affiliations:** 1 Department of Medical Biochemistry and Microbiology, Uppsala University, Uppsala, Sweden; 2 Biologisk Myggkontroll, Nedre Dalälvens Utvecklings AB, Gysinge, Sweden; 3 Princeton University Department of Ecology and Evolutionary Biology, Princeton, New Jersey, USA; 4 Zuckerman Mind Brain Behavior Institute, Columbia University, New York, New York, USA; 5 Tree of Life, Wellcome Sanger Institute, Hinxton, England, UK; 6 University of Cambridge Department of Genetics, Cambridge, England, UK

**Keywords:** Culex pipiens, northern house mosquito, genome sequence, chromosomal

## Abstract

We present a genome assembly from an individual female *Culex pipiens* sensu stricto (the northern house mosquito; Arthropoda; Insecta; Diptera; Culicidae), from a wild population in Sweden. The genome sequence is 533 megabases in span. Most of the assembly is scaffolded into three chromosomal pseudomolecules. The complete mitochondrial genome was also assembled and is 15.6 kilobases in length.

## Species taxonomy

Animalia; Arthropoda; Insecta; Diptera; Culicidae; Culex; *Culex pipiens*; Linnaeus, 1758 (NCBI taxid:7174).

## Background

The northern house mosquito *Culex pipiens* (Linnaeus, 1758) is a cosmopolitan species found in temperate zones across both the northern and southern hemispheres. It is a member of the *Culex pipiens* species complex, which also includes the widespread tropical/subtropical species *Cx. quinquefasciatus*, an East Asian species *Cx. pallens*, and two Australian endemics, *Cx. australicus*, and *Cx. globocoxitus* ( Vinogradova, 2000). All five species are morphologically similar, often only distinguishable by male genitalia. *Cx. pipiens* forms latitudinal hybrid zones with *Cx. quinquefasciatus* where they come into contact in North America and Asia ( Fonseca *et al.*, 2009; Kothera *et al.*, 2009). The only place where the two cosmopolitan species appear able to coexist without hybridization is southern Africa ( Cornel *et al.*, 2003).

*Cx. pipiens* displays surprising ecological diversity. The habits of southern African populations are not well understood, but northern populations comprise two morphologically indistinguishable ecotypes or forms, termed *pipiens* and *molestus* ( Farajollahi *et al.*, 2011; Haba & McBride, 2022; Vinogradova, 2000). Form *pipiens* females diapause in winter and primarily bite birds. They represent important bridge vectors of West Nile Virus, a bird virus for which humans are dead-end hosts. Form *molestus* females breed year-round. They can produce a first clutch of eggs without a blood meal (autogeny) but also readily bite humans and other mammals. Form *molestus* canonically breeds in urban below ground environments, such as subways, cellars, and cesspits. However, they also thrive above ground in Mediterranean climates, including the Mediterranean basin itself, as well as Argentina and Australia, where they were introduced quite recently ( Chevillon *et al.*, 1995; Di Luca *et al.*, 2016; Kassim *et al.*, 2013; Mitchell & Darsie, 1985; Urbanelli *et al.*, 1981). Form *molestus* served as the primary vector of lymphatic filariasis in Egypt until the pathogen was locally eradicated in the early 2000s ( Ramzy *et al.*, 2019).

The abundance and public health importance of *Cx. pipiens* has led to many genetic studies. Early studies were aimed at distinguishing ecotypes with allozymes and microsatellites ( Byrne & Nichols, 1999; Fonseca *et al.*, 2004; Urbanelli *et al.*, 1981). A single-locus PCR assay has also been developed to reliably separate *pipiens* and *molestus* at the population (but not individual) level in colder northern latitudes ( Bahnck & Fonseca, 2006). Most recently, authors have begun to apply genome-wide markers, such as AFLPs ( Gomes *et al.*, 2015), or high throughput sequencing approaches, such as RNAseq and gene-based capture ( Aardema *et al.*, 2020; Aardema *et al.*, 2022). Together this work has revealed complex population structure and extensive geographic variation that is still not well understood. Long read chromosome-level assemblies with gene annotations are available for *Cx. quinquefasciatus* (GCF_015732765.1) ( Ryazansky *et al.*, 2024), *Cx. pallens* (GCF_016801865.2), and *Cx. pipiens* f. *molestus* (GCA_024516115.1) ( Liu *et al.*, 2023). Here, we present a chromosomally complete genome sequence for *Culex pipiens* f. *pipiens* using a single female specimen collected in diapause (hibernating) in a food-cellar in Uppsala, Sweden.

## Genome sequence report

The genome was sequenced from a single female *Culex pipiens* mosquito collected in April 2021 in Uppsala, Sweden (59.75, 17.51). A total of 19-fold coverage per haplotype in Pacific Biosciences single-molecule HiFi long reads (N50 12.609 kb for low input library and 9.222 kb for ultra-low input library) were generated. Primary assembly contigs were scaffolded with chromosome conformation Hi-C data from a female mosquito caught in the same location in April 2021. Manual assembly curation corrected 187 missing joins or misjoins and removed 152 haplotypic duplications, reducing the scaffold number by 75.8% and reducing the assembly size by 19.3%.

**Figure 1.**
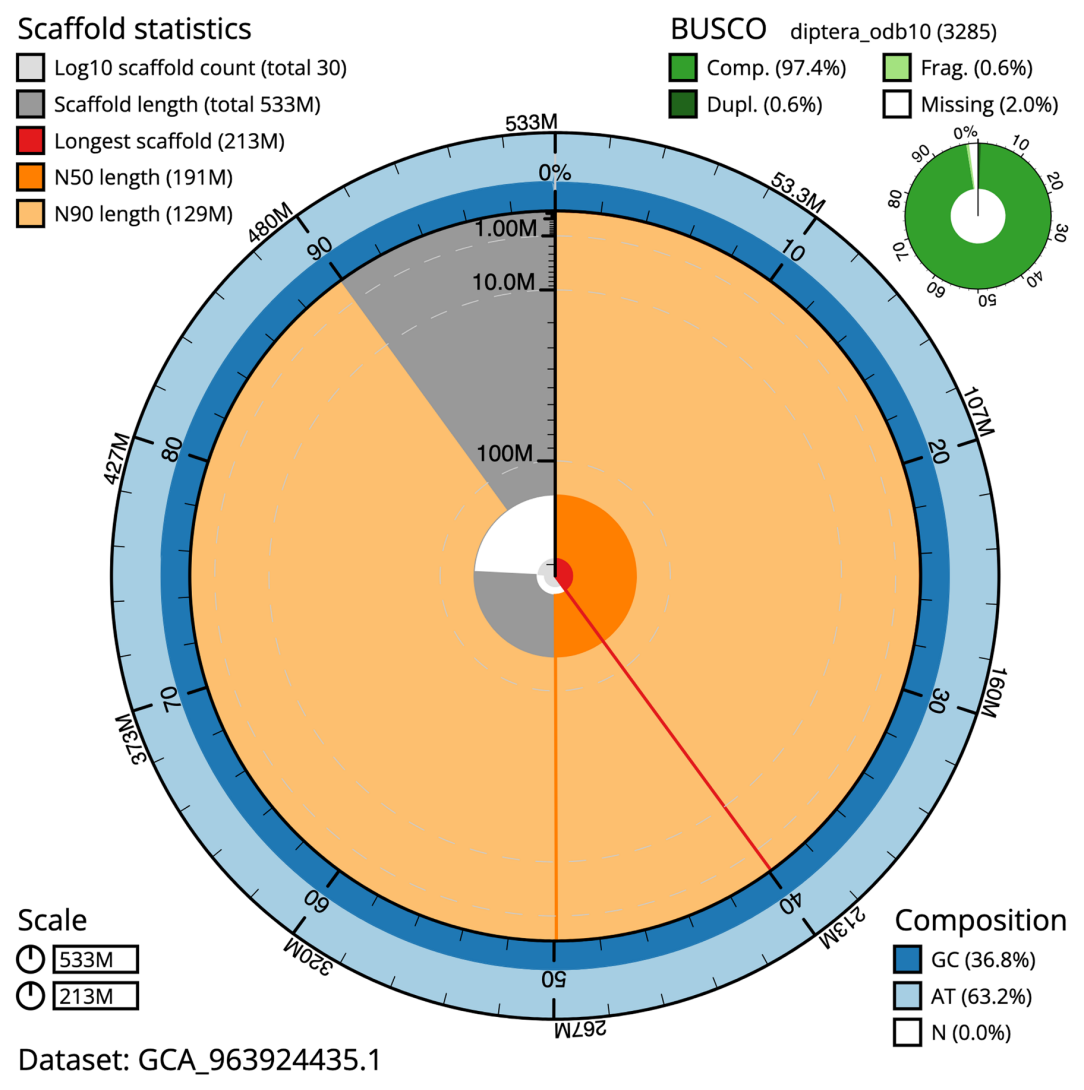
Snail plot summary of assembly statistics for assembly idCulPipi1.1. The main plot is divided into 1,000 bins around the circumference with each bin representing 0.1% of the 533,168,032 bp assembly. The distribution of sequence lengths is shown in dark grey with the plot radius scaled to the longest sequence present in the assembly (213,114,244 bp, shown in red). Orange and pale-orange arcs show the N50 and N90 sequence lengths (190,882,370 and 128,522,754 bp), respectively. The pale grey spiral shows the cumulative sequence count on a log scale with white scale lines showing successive orders of magnitude. The blue and pale-blue area around the outside of the plot shows the distribution of GC, AT and N percentages in the same bins as the inner plot. A summary of complete, fragmented, duplicated and missing BUSCO genes in the diptera_odb10 set is shown in the top right. An interactive version of this figure is available at https://blobtoolkit.genomehubs.org/view/idCulPipi1.1/dataset/GCA_963924435.1/snail.

**Figure 2.**
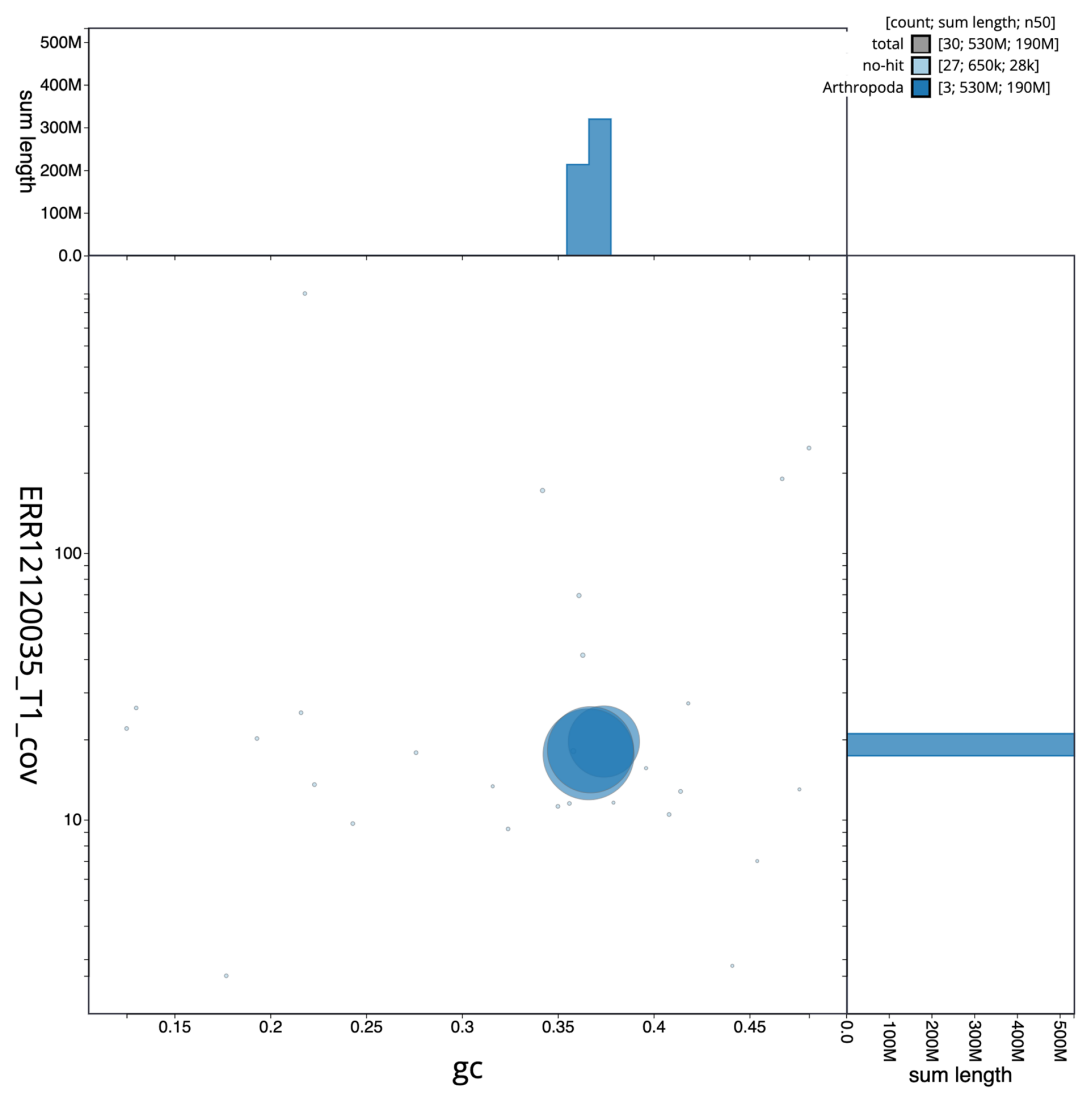
Blob plot of base coverage in a subset of idCulPipi1 pacbio reads (ERR12120035) against GC proportion for *Cx. pipiens* assembly idCulPipi1. Chromosomes are coloured by phylum. Circles are sized in proportion to chromosome length. Histograms show the distribution of chromosome length sum along each axis. An interactive version of this figure is available at https://blobtoolkit.genomehubs.org/view/idCulPipi1.1/dataset/GCA_963924435.1/blob.

**Figure 3.**
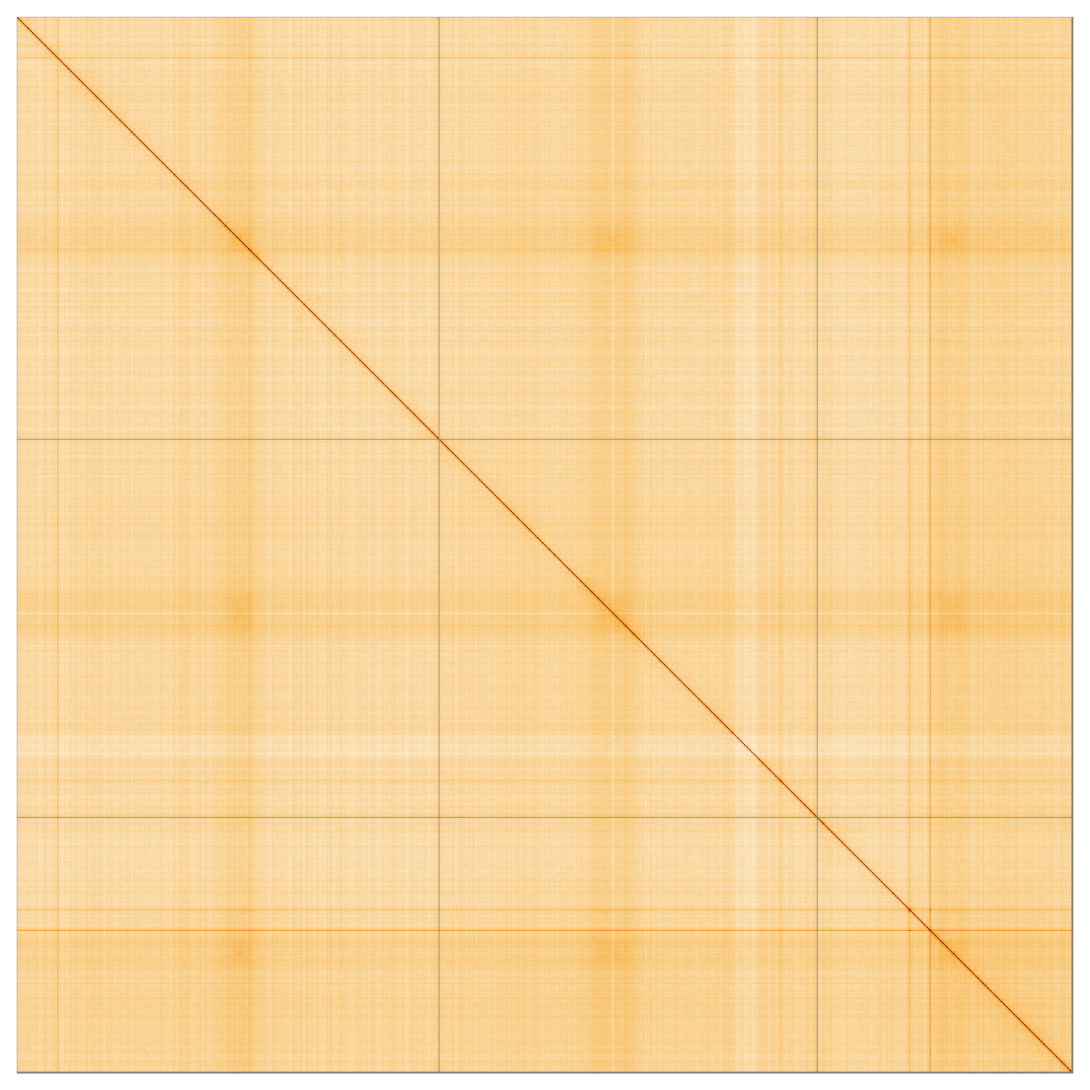
Hi-C contact map for genome assembly of *Culex pipiens, idCulPipi1.1*. Visualised in HiGlass. Chromosomes order: 2, 3, 1, then remaining scaffolds. The interactive Hi-C map can be viewed at https://genome-note-higlass.tol.sanger.ac.uk/l/?d=Sjyb5raPQzqn3xHqRpD_YQ.

**Figure 4.**
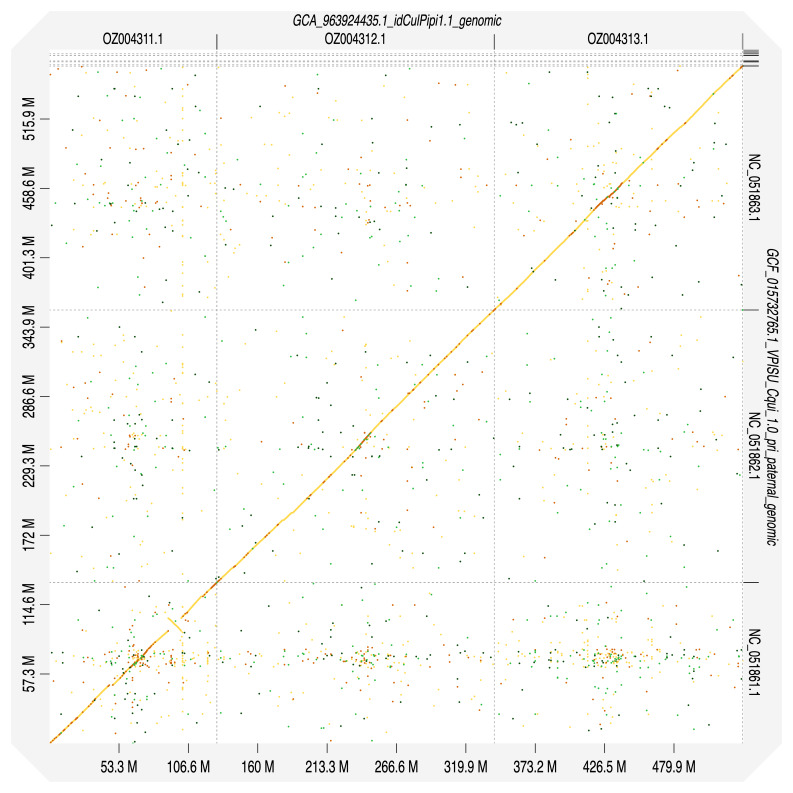
Alignment dotplot between genome assemblies of *Culex pipiens* (idCulPipi1.1) and *Cx. quinquefasciatus*, VPISU_Cqui_1.0_pri_paternal (JHB strain). Visualised in DGenies.

The final assembly has a total length of 533 Mb in 29 sequence scaffolds with a scaffold N50 of 190.9 Mb ( Table 1). The snail plot in Figure 1 provides a summary of the assembly statistics, while the distribution of assembly scaffolds on GC proportion and coverage is shown in Figure 2. 99.88% of the assembly sequence was assigned to three chromosomal-level scaffolds ( Figure 3; Table 2). Chromosomes were numbered and oriented using synteny to the *Culex quinquefasciatus* JHB strain assembly VPISU_Cqui_1.0_pri_paternal ( Ryazansky *et al.*, 2024) (accession GCF_015732765.1) ( Figure 4). The assembly has a BUSCO 5.3.2 ( Simão *et al.*, 2015) completeness of 97.4% using the diptera_odb10 reference set. While not fully phased, the assembly deposited is of one haplotype and also includes the circular mitochondrial genome. Contigs corresponding to the second haplotype have also been deposited.

**Table 1.** Genome data for *Culex pipiens*, idCulPipi1.1.

*Project accession data*	
Assembly identifier	idCulPipi1.1
Species	*Culex pipiens*
Specimen	idCulPipi1
NCBI taxonomy ID	7175
BioProject	PRJEB67967
BioSample ID	ERS14890764
Isolate information	female, whole organism
*Raw data accessions*	
PacificBiosciences SEQUEL II	ERR12120034, ERR12120035
Hi-C Illumina	ERR12411010
*Genome assembly*	
Assembly accession	GCA_963924435.1
*Accession of alternate haplotype*	GCA_963924485.1
Span (Mb)	533.2
Number of contigs	326
Contig N50 length (Mb)	3.3
Number of scaffolds	29
Scaffold N50 length (Mb)	190.9
Longest scaffold (Mb)	213.1
BUSCO * genome score	C:97.4%[S:96.8%,D:0.6%],F:0.6%,M:2.0%,n:3285

* BUSCO scores based on the diptera_odb10 BUSCO set using BUSCO 5.3.2. C=complete [S=single copy, D=duplicated], F=fragmented, M=missing, n=number of orthologues in comparison. A full set of BUSCO scores is available at https://blobtoolkit.genomehubs.org/view/idCulPipi1.1/dataset/GCA_963924435.1/busco.

**Table 2.** Chromosomal pseudomolecules in the genome assembly of *Culex pipiens*, idCulPipi1.1.

INSDC accession	Chromosome	Size (Mb)	Count	Gaps
**OZ004311.1**	**1**	**128.523**	**1**	**92**
**OZ004312.1**	**2**	**213.114**	**1**	**98**
**OZ004313.1**	**3**	**190.882**	**1**	**106**
**OZ004314.1**	**MT**	**0.016**	**1**	**0**
	**1 Unlocalised**	**0.053**	**1**	**0**
	**Unplaced**	**0.580**	**25**	**1**

**Figure 5.**
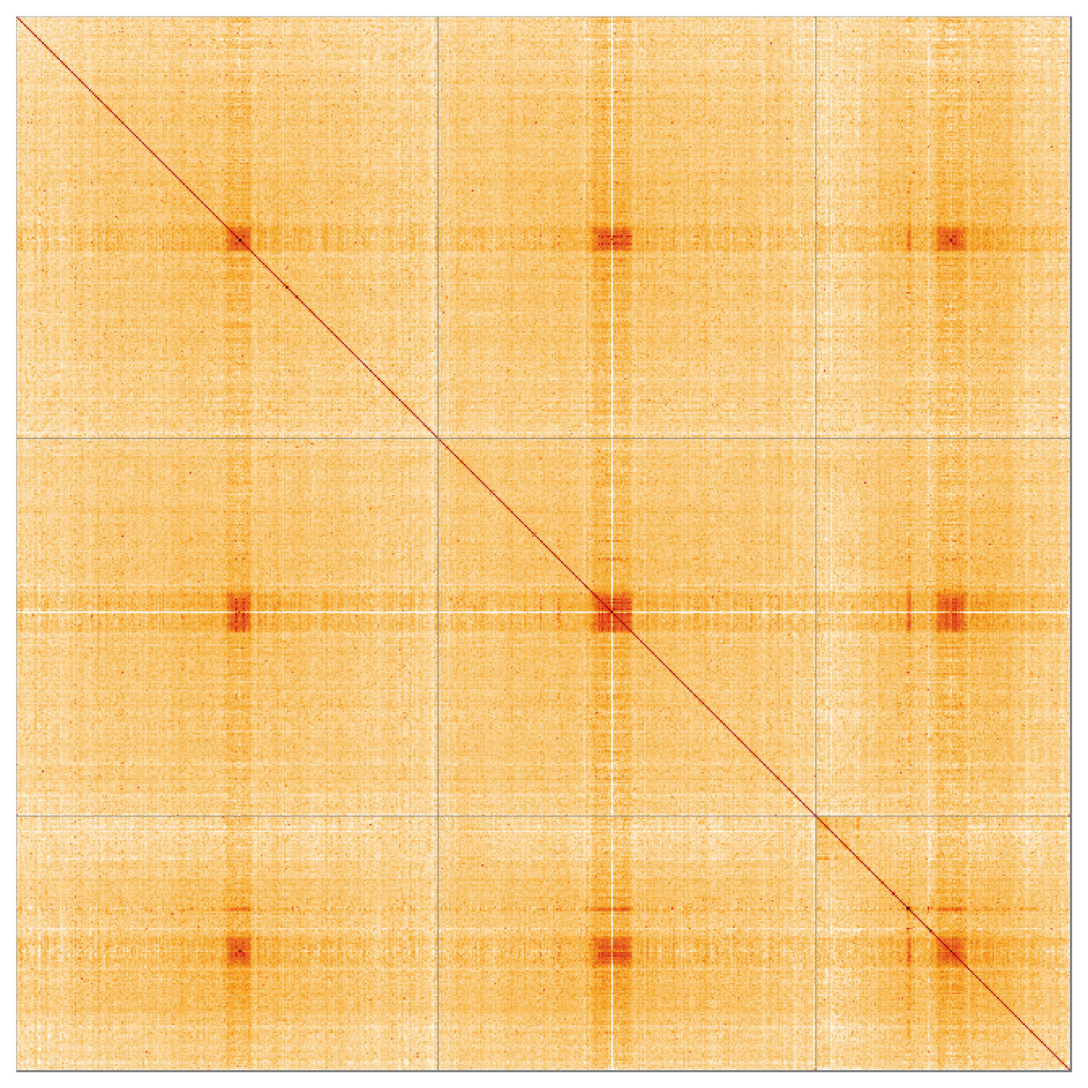
Sequence similarity heatmap for genome assembly of *Culex pipiens, idCulPipi1.1*. Produced with StainedGlass, visualised in HiGlass. Chromosomes order: 2, 3, 1 - followed by the remaining scaffolds. Darker colours represent higher sequence similarity, notably at pericentric heterochromatin.

Chromosome arms, candidate centromere sequences, and the rDNA region were delineated based on the presence of characteristic tandem repeat arrays ( Figure 5; Table 3). Candidate centromere regions were represented by complex tandem repeat blocks with significant sequence similarity between all three chromosomes. The largest cluster of rDNA genes was located on chromosome arm 1p between 56.905-57.009 Mbp, some 1.5 Mbp away from the predicted M-locus between 58.9-61.9 Mbp in chromosome 1 of *Cx. quinquefasciatus* ( Ryazansky *et al.*, 2024) - interestingly, the majority of predicted M-locus has homologous sequences in our female *Cx. pipiens* assembly.

**Table 3.** Chromosome arms in the genome assembly of *Culex pipiens*, idCulPipi1.1.

Chromosome	Start	End	Chromosome arm
**1**	**1**	**68,424,560**	**1p**
**1**	**68,488,620**	**128,522,754**	**1q**
**2**	**1**	**112,539,609**	**2p**
**2**	**113,453,184**	**213,114,244**	**2q**
**3**	**1**	**87,925,989**	**3p**
**3**	**88,082,842**	**190,882,370**	**3q**

## Methods

### Sample acquisition and nucleic acid extraction

Hibernating *Culex pipiens* specimens were collected from Uppsala, Sweden (59.75, 17.51) by Jenny Hesson in April 2021. A single female idCulPipi1 was used for Pacific BioSciences, another female idCulPipi2 was used for Arima Hi-C. Mosquitoes were prepared using the “squish method” ( Teltscher & Lawniczak, 2023) and shipped at room temperature in ethanol overnight to the UK.

For high molecular weight (HMW) DNA extraction, one whole female insect (idCulPipi1) was disrupted by manual grinding with a blue plastic pestle in Qiagen MagAttract lysis buffer and then extracted using the Qiagen MagAttract HMW DNA extraction kit with two minor modifications including halving volumes recommended by the manufacturer due to small sample size and running two elution steps of 100 μl each to increase DNA yield ( Teltscher *et al.*, 2023). The quality of the DNA was evaluated using an Agilent FemtoPulse to ensure that most DNA molecules were larger than 30 kb, and preferably > 100 kb.The average fragment size for this sample was 200 kb. The DNA yield obtained for this sample was 703 ng. DNA was sheared using a Diagenode Megaruptor 3 as follows: the total volume of the DNA extract was first sheared at speed 30 (low input (LI) library). An aliquot was then removed (54 µL), topped up with Qiagen Elution buffer to a volume of 100 µL and sheared again at speed 33 (ultra low input (ULI) library). The average fragment size obtained for the LI library and the ULI library were 13.5 kb and 13.6 kb respectively. Sheared DNA was purified using AMPure PB beads with a 0.6X ratio of beads to sample to remove the shorter fragments and concentrate the DNA sample. The concentration and quality of the sheared and purified DNA was assessed using a Nanodrop spectrophotometer and Qubit Fluorometer with the Qubit dsDNA High Sensitivity Assay kit. Fragment size distribution was evaluated by running the sheared and cleaned sample on the FemtoPulse system once more. For the LI library, the average fragment size obtained was 16.5 kb and the DNA yield was 354 ng. For the ULI library, the average fragment size obtained was 13.9 kb and the DNA yield was 125 ng. These libraries lost 50% and 38% of DNA through the process of shearing and SPRI respectively.

For Hi-C data generation, a separate unrelated female mosquito specimen (idCulPipi2) was used as input material for the Arima V2 Kit according to the manufacturer’s instructions for animal tissue. This approach of using another individual was taken in order to enable all material from a single specimen to contribute to the PacBio data generation given we were not able to meet the minimum required HMW DNA and also save tissue for Hi-C from a single specimen.

### Sequencing

We prepared libraries as per the PacBio procedure and checklist for SMRTbell Libraries using Express TPK 2.0 with low DNA input. Coverage with the single individual low input library was not sufficient so we topped up this library with an Ultra Low Input library using additional DNA from the same mosquito. Sequencing complexes were made using Sequencing Primer v4 and DNA Polymerase v2.0. Sequencing was carried out on the Sequel II system with 24-hour run time and 2-hour pre-extension. For Hi-C data generation, following the Arima HiC V2 reaction, samples were processed through Library Preparation using a NEB Next Ultra II DNA Library Prep Kit and sequenced aiming for 100x depth. Sequencing was performed by the Scientific Operations core at the Wellcome Sanger Institute on Pacific Biosciences SEQUEL II (HiFi), Illumina NovaSeq 6000 (Hi-C).

### Genome assembly

The HiFi reads were first assembled using Hifiasm ( Cheng *et al.*, 2021) with the --primary option. Haplotypic duplications were identified and removed with purge_dups ( Guan *et al.*, 2020). The Hi-C reads were mapped to the primary contigs using bwa-mem2 ( Vasimuddin *et al.*, 2019). The contigs were further scaffolded using the provided Hi-C data ( Rao *et al.*, 2014) in yahs ( Zhou *et al.*, 2023). using the --break option for handling potential misassemblies. The scaffolded assemblies were evaluated using Gfastats ( Formenti *et al.*, 2022), BUSCO ( Manni *et al.*, 2021) and MERQURY.FK ( Rhie *et al.*, 2020). The mitochondrial genome was assembled using MitoHiFi ( Uliano-Silva *et al.*, 2021), which performs annotation using MitoFinder ( Allio *et al.*, 2020) and uses these annotations to select the final mitochondrial contig and to ensure the general quality of the sequence.

### Assembly curation

The assembly was decontaminated using the Assembly Screen for Cobionts and Contaminants (ASCC) pipeline (article in preparation). Flat files and maps used in curation were generated in TreeVal ( Pointon *et al.*, 2023). Manual curation was primarily conducted using PretextView ( Harry, 2022), with additional insights provided by JBrowse2 ( Diesh *et al.*, 2023) and HiGlass ( Kerpedjiev *et al.*, 2018). Scaffolds were visually inspected and corrected as described by ( Howe *et al.*, 2021). Any identified contamination, missed joins, and mis-joins were corrected, and duplicate sequences were tagged and removed. The curation process is documented at https://gitlab.com/wtsi-grit/rapid-curation (article in preparation).

### Evaluation of final assembly

A HiGlass map was created to show the final assembly. The Hi-C reads are aligned using using bwa-mem2 ( Vasimuddin *et al.*, 2019) and the alignment files are combined with SAMtools ( Danecek *et al.*, 2021). The Hi-C alignments are converted into a contact map using BEDTools ( Quinlan & Hall, 2010) and the Cooler tool suite ( Abdennur & Mirny, 2020). The contact map is visualised in HiGlass ( Kerpedjiev *et al.*, 2018).

The blobtoolkit pipeline ( Muffato *et al.*, 2024). is a Nextflow ( Di Tommaso *et al.*, 2017) port of the previous Snakemake Blobtoolkit pipeline ( Challis *et al.*, 2020). It aligns the PacBio reads in SAMtools and minimap2 ( Li, 2018) and generates coverage tracks for regions of fixed size. In parallel, it queries the GoaT database ( Challis *et al.*, 2023) to identify all matching BUSCO lineages to run BUSCO ( Manni *et al.*, 2021). For the three domain-level BUSCO lineages, the pipeline aligns the BUSCO genes to the UniProt Reference Proteomes database ( UniProt Consortium, 2023) with DIAMOND blastp ( Buchfink *et al.*, 2021). The genome is also divided into chunks according to the density of the BUSCO genes from the closest taxonomic lineage, and each chunk is aligned to the UniProt Reference Proteomes database using DIAMOND blastx. Genome sequences without a hit are chunked using seqtk and aligned to the NT database with blastn ( Altschul *et al.*, 1990). The blobtools suite combines all these outputs into a blobdir for visualisation.

The genome assembly and evaluation pipelines were developed using nf-core tooling ( Ewels *et al.*, 2020) and MultiQC ( Ewels *et al.*, 2016), relying on the Conda package manager, the Bioconda initiative ( Grüning *et al.*, 2018), the Biocontainers infrastructure ( da Veiga Leprevost *et al.*, 2017), as well as the Docker ( Merkel, 2014) and Singularity ( Kurtzer *et al.*, 2017) containerisation solutions.

Table 4 contains a list of all software tool versions used, where appropriate.

**Table 4.** Software tools used.

Software tool	Version	Source
BEDTools	2.30.0	https://github.com/arq5x/bedtools2
BLAST	2.14.0	ftp://ftp.ncbi.nlm.nih.gov/blast/executables/blast+/
BlobToolKit	4.3.7	https://github.com/blobtoolkit/blobtoolkit
BUSCO	5.4.3 and 5.5.0	https://gitlab.com/ezlab/busco
bwa-mem2	2.2.1	https://github.com/bwa-mem2/bwa-mem2
Cooler	0.8.11	https://github.com/open2c/cooler
D-GENIES	1.4	https://github.com/genotoul-bioinfo/dgenies
DIAMOND	2.1.8	https://github.com/bbuchfink/diamond
fasta_windows	0.2.4	https://github.com/tolkit/fasta_windows
FastK	427104ea91c78c3b8b8b49f1a7d6bbeaa869ba1c	https://github.com/thegenemyers/FASTK
Gfastats	1.3.6	https://github.com/vgl-hub/gfastats
GoaT CLI	0.2.5	https://github.com/genomehubs/goat-cli
Hifiasm	0.19.5-r587	https://github.com/chhylp123/hifiasm
HiGlass	1.11.6	https://github.com/higlass/higlass
Merqury.FK	d00d98157618f4e8d1a9190026b19b471055b22e	https://github.com/thegenemyers/MERQURY.FK
minimap2	2.24	https://github.com/lh3/minimap2
MitoHiFi	2	https://github.com/marcelauliano/MitoHiFi
MultiQC	1.14, 1.17, and 1.18	https://github.com/MultiQC/MultiQC
NCBI Datasets	15.12.0	https://github.com/ncbi/datasets
Nextflow	23.04.0-5857	https://github.com/nextflow-io/nextflow
PretextView	0.2.5	https://github.com/sanger-tol/PretextView
purge_dups	1.2.5	https://github.com/dfguan/purge_dups
samtools	1.16.1, 1.17, and 1.18	https://github.com/samtools/samtools
sanger-tol/ascc	-	https://github.com/sanger-tol/ascc
sanger-tol/blobtoolkit	0.6.0	https://github.com/sanger-tol/blobtoolkit
Seqtk	1.3	https://github.com/lh3/seqtk
Singularity	3.9.0	https://github.com/sylabs/singularity
StainedGlass	0.5	https://github.com/mrvollger/StainedGlass
TreeVal	1.0.0	https://github.com/sanger-tol/treeval
YaHS	1.2a.2	https://github.com/c-zhou/yahs

### Ethics/compliance issues

The genetic resources accessed and utilised under this project were done so in accordance with the UK ABS legislation (Nagoya Protocol (Compliance) (Amendment) (EU Exit) Regulations 2018 (SI 2018/1393)) and the national ABS legislation within the country of origin, where applicable.

## Data Availability

European Nucleotide Archive: *Culex pipiens* genome assembly, idCulPipi1. Accession number PRJEB67967; https://identifiers.org/bioproject/PRJEB67967. The genome sequence is released openly for reuse. All raw sequence data and the assembly have been deposited in INSDC databases. Raw data and assembly accession identifiers are reported in Table 1.

## Author information

Members of the Sanger Scientific Operations: DNA Pipelines collective are listed here: https://doi.org/10.5281/zenodo.4790456.
