# Supplementary material for: A chromosomal reference genome sequence for the northern house mosquito, Culex pipiens form pipiens, Linnaeus, 1758
Source: Wellcome Open Res. 2025 Feb 26;10:107. [Version 1] doi: 10.12688/wellcomeopenres.23767.1 (PMC11923538; doi:10.12688/wellcomeopenres.23767.1)
Supplement: Peer review history

# Reviewer report: Reviewer response for version 1

**DOI:** 10.21956/wellcomeopenres.26215.r119683

**Published:** 2025-03-19

**Authors:** Anthony Bayega

**Affiliations:** 1 McGill University, Montreal, Canada

Hesson et al. provide a chromosomal-level genome assembly of *Culex pipiens *form *pipiens*. I commend them for their efforts. My comments follow below:

 1. In Figure 3, the label on the Y-axis which reads “ERR1…” could be changed to something more informative to help the reader make sense of this figure.

 2. It is commendable that the group has attempted to decontaminate the assembly of Cobionts.

 Overall, the authors provide a high-quality genome and also assign 99.88% of it to chromosomes. Although much work remains to order the scaffolds, fully phase the contigs and scaffolds, and complete the gaps and also structurally and functionally annotate the genome, the current work will indeed be valuable to the whole community. I therefore recommend the indexing of this genome so that this resource becomes widely accessible to the scientific community.

Are sufficient details of methods and materials provided to allow replication by others?

Yes

Is the rationale for creating the dataset(s) clearly described?

Yes

Are the datasets clearly presented in a useable and accessible format?

Yes

Are the protocols appropriate and is the work technically sound?

Yes

Reviewer Expertise:

Genomics and genetics

I confirm that I have read this submission and believe that I have an appropriate level of expertise to confirm that it is of an acceptable scientific standard.

# Reviewer report: Reviewer response for version 1

**DOI:** 10.21956/wellcomeopenres.26215.r119677

**Published:** 2025-03-13

**Authors:** Marcus Stensmyr

**Affiliations:** 1 Lund University, Lund, Sweden

A chromosome length assembly of the northern house mosquito genome is a most welcome addition to the field. The genome is 533MB in length, assembled into three main scaffolds (plus the mitochondrial genome), and is 97.4% complete. The genome has been sequenced and assembled using state-of-the-art methods. It would have been even better if the authors had included annotation of the genome, which I'm a bit surprised is missing.

Are sufficient details of methods and materials provided to allow replication by others?

Yes

Is the rationale for creating the dataset(s) clearly described?

Yes

Are the datasets clearly presented in a useable and accessible format?

Partly

Are the protocols appropriate and is the work technically sound?

Yes

Reviewer Expertise:

Diptera neurogenetics and genomics

I confirm that I have read this submission and believe that I have an appropriate level of expertise to confirm that it is of an acceptable scientific standard.
